# Current clinical and research practices on frontotemporal dementia in Brazil: a national survey

**DOI:** 10.1055/s-0043-1771173

**Published:** 2023-07-26

**Authors:** Leonardo Cruz de Souza, Sonia Maria Dozzi Brucki, Lucas Porcello Schilling, Letícia Costa da Silva, Leonel Tadao Takada, Valéria Santoro Bahia, Breno José Alencar Pires Barbosa, Marcio Luiz Figueredo Balthazar, Norberto Anizio Ferreira Frota, Ricardo Nitrini, Paulo Caramelli, Jerusa Smid

**Affiliations:** 1 Universidade Federal de Minas Gerais, Departamento de Clínica Médica, Belo Horizonte MG, Brazil.; 2 Universidade Federal de Minas Gerais, Faculdade de Medicina, Departamento de Clínica Médica, Grupo de Pesquisa em Neurologia Cognitiva e do Comportamento, Belo Horizonte MG, Brazil.; 3 Universidade de São Paulo, Faculdade de Medicina, Departamento de Neurologia, Grupo de Neurologia Cognitiva e do Comportamento, São Paulo SP, Brazil.; 4 Pontifícia Universidade do Rio Grande do Sul, Escola de Medicina, Serviço de Neurologia, Porto Alegre RS, Brazil.; 5 Pontifícia Universidade do Rio Grande do Sul, Instituto do Cérebro do Rio Grande do Sul, Porto Alegre RS, Brazil.; 6 Pontifícia Universidade do Rio Grande do Sul, Programa de Pós-Graduação em Gerontologia Biomédica, Porto Alegre RS, Brazil.; 7 Universidade Cidade de São Paulo, São Paulo SP, Brazil.; 8 Universidade Federal de Pernambuco, Centro de Ciências Médicas, Área Acadêmica de Neuropsiquiatria, Recife PE, Brazil.; 9 Universidade Estadual de Campinas, Departamento de Neurologia, São Paulo SP, Brazil.; 10 Hospital Geral de Fortaleza, Serviço de Neurologia, Fortaleza CE, Brazil.; 11 Universidade de Fortaleza (UNIFOR), Fortaleza CE, Brazil.

**Keywords:** Frontotemporal Dementia, Aging, Dementia, Demência Frontotemporal, Envelhecimento, Demência

## Abstract

**Background**  Frontotemporal dementia (FTD) is a frequent cause of young-onset dementia and represents a major challenge for the diagnosis and clinical management. It is essential to evaluate the difficulties faced by physicians on the diagnostic workup and on patient care.

**Objective**  The aim of this study was to investigate the current practices and the local limits on the diagnosis and management of FTD in Brazil.

**Methods**  We elaborated an online survey, composed of 29 questions and divided in four parts, comprising questions about existing health facilities, clinical practices related to FTD, and suggestions to increment the national research on FTD. The invitation to participate was sent by email to all neurologists affiliated to the Brazilian Academy of Neurology ( *n*  = 3658), and to all physicians who attended the XII Meeting of Researchers on Alzheimer's disease, in 2019 ( *n*  = 187). The invitation was also diffused through social media.

**Results**  256 Brazilian physicians answered the questionnaire. The three most relevant disorders for the differential diagnosis of FTD were Alzheimer's disease (AD) ( *n*  = 211), bipolar disorder ( *n*  = 117) and dementia with Lewy bodies ( *n*  = 92). Most respondents (125/256) reported the difficulty in performing genetic testing as the main limit in the diagnostic of FTD. 93% and 63% of participants considered that the assessment of social cognition and AD CSF biomarkers are useful for the diagnosis of FTD, respectively.

**Conclusions**  The present study may provide valuable insights for the medical education and clinical training of physicians, and to foster future research on FTD in Brazil.

## Resumo

**Antecedentes**  A demência frontotemporal (DFT) é causa frequente de demência pré-senil e representa um desafio em termos de diagnóstico e de manejo clínico. É essencial avaliar as dificuldades existentes na propedêutica e nos cuidados médicos.

**Objetivo**  Investigar as práticas médicas e as dificuldades para diagnóstico e manejo da DFT no Brasil.

**Métodos**  Elaborou-se um questionário online, composto de 29 questões, divididas em quatro partes, com perguntas sobre infraestrutura existente, práticas clínicas relacionadas à DFT e sugestões para desenvolver a pesquisa nacional na área. O convite para participação foi enviado por e-mail a todos neurologistas afiliados à Academia Brasileira de Neurologia ( *n*  = 3658), e aos médicos que participaram da XII Reunião de Pesquisadores de Doença de Alzheimer, em 2019 ( *n*  = 187). O convite também foi divulgado através de mídias sociais.

**Resultados**  256 médicos brasileiros responderam o questionário. Os três principais diagnósticos diferenciais de DFT foram doença de Alzheimer ( *n*  = 211), transtorno bipolar ( *n*  = 117) e demência com corpos de Lewy ( *n*  = 92). A maior parte dos respondedores (125/256) apontou a dificuldade em realizar testagem genética como o maior limite no diagnóstico de DFT. 93% e 63% dos respondedores indicaram que a avaliação de cognição social e o uso de biomarcadores liquóricos de doença de Alzheimer são úteis no diagnóstico de DFT, respectivamente.

**Conclusões**  Estes resultados devem ser considerados na educação e treinamento médicos, e no desenvolvimento da pesquisa brasileira em DFT.

## INTRODUCTION

 Since the last decades, Brazil has been facing population aging, with impacts on demographics, economics, and on the health care system. The prevalence of age-related disorders, such as dementias, is increasing and represents one of the most frequent causes of mortality. 1 In particular, young-onset dementias represent a major challenge for the clinical management, as specialized health professionals and adequate structures are lacking, 2 thus increasing the burden of patients and families. 

 Frontotemporal dementia (FTD) is the second most frequent cause of young-onset dementia, following Alzheimer's disease (AD). 3 FTD is actually defined as a clinical syndrome with three phenotypes: the behavioral variant (bvFTD) and two language variants, nonfluent/agrammatic primary progressive aphasia (nf/aPPA) and semantic variant (svPPA). 3 The behavioral variant is the most common presentation, and manifests with variable degrees of personality changes and behavioral disorders. 4 The language variants manifest major language disturbance as early symptoms: while patients with nf/aPPA present with effortful speech or agrammatism, svPPA patients exhibit reduced single-word comprehension associated to impaired naming in confrontation tests. 5

 Data from high-income countries suggest that the prevalence of FTD is estimated as 15–22 cases/100.000, with higher incidence among individuals from 45 to 64 years-old. 6 No study specifically addressed the prevalence of FTD in Brazil, but epidemiological surveys of dementia found a prevalence of 0.18%, in individuals older than 65 years-old. 7

 Although FTD is not a frequent disorder, it should be pointed out that FTD and other young-onset dementias represent a major challenge for medical care, especially in Brazil and in other low- and middle-income countries, where medical facilities and specialized health care teams are insufficient. 2 Of note, FTD is associated with faster decline and higher caregiver burden, compared with AD, 3 thus requiring more health care resources. 

 In this scenario, it is essential to ascertain the difficulties Brazilian physicians face on the diagnostic workup, and the local struggles in the care of FTD patients as well. National surveys may provide valuable information for improving the public awareness, the clinical care and the research in FTD. 8 9 This study aimed to investigate current practices on the diagnosis and management of FTD in Brazil. Moreover, we also assessed the existing facilities and we investigated which are the main limits for clinical practice and research development in the field of FTD, from the perspective of physicians. 

## METHODS

 The questionnaire was elaborated by the Scientific Department of Cognitive Neurology and Aging from the Brazilian Academy of Neurology. Questions from similar studies 8 9 were also included in the present survey. 

 The questionnaire ( **Supplementary Material** - https://www.arquivosdeneuropsiquiatria.org/wp-content/uploads/2023/07/ANP-2023.0016-Supplementary-Material.pdf ) was created on an online platform (Google Forms®) and was available for answering from 9 ^th^ November 2020 to 26 ^th^ January 2021. The survey comprised 29 questions, divided in four parts. The total estimated time to respond to it was five minutes. The first part collected general information from the respondent: city/state, affiliation, medical specialty, composition of the health team, number of FTD patients followed by the participant, annual number of new diagnosis of FTD, number of genetic cases, experience in clinical research in FTD, and facilities (data bank, neuroimaging bank, biobank, brain bank). This first part also addressed how the participant conducted clinical investigation of suspected FTD cases, by presenting questions about the clinical interview (which are the key questions usually asked on the medical interview) and the cognitive/behavioral assessment. 

The second part presented questions about clinical management. There were questions about the availability of support groups for family and caregivers, and previous experience with clinical trials. This part also addressed the participant's experience with pharmacological treatment of FTD (use of antipsychotics, antiseizure medication [ASM], serotonin reuptake inhibitors, trazodone and psychostimulants).

The third part proposed questions regarding personal opinion on diagnostic and management of FTD. The questionnaire asked about which are the participant's main limit in the diagnostic procedure of FTD (difficulty to perform formal neuropsychological testing, genetic investigation, CSF biomarkers, structural or functional neuroimaging) and which are the main causes of misdiagnosis of FTD on the participant's view. This part also asked whether the participant considers CSF biomarkers for AD and social cognition tests to be useful in the diagnostic investigation of FTD, and whether he/she considers episodic memory impairment a valuable feature to distinguish FTD from AD.

Finally, the fourth part collected participant's suggestions to improve clinical care and to improve FTD research, by making questions about the need of new epidemiological studies, new cognitive/behavioral tools, facilitation of genetic investigation, and the proposal of a common national protocol for research purposes.

 The invitation to participate was sent by email to all neurologists affiliated to the Brazilian Academy of Neurology ( *n*  = 3658), and to all physicians who attended the XII Meeting of Researchers on Alzheimer's disease, in 2019 ( *n*  = 187), a multidisciplinary meeting. In addition, the invitation was also diffused to physicians through social media. 

This study was approved by the Executive Committee of the Brazilian Academy of Neurology. All participants provided formal consent to this study. As a matter of confidentiality, all answers were processed anonymously.

## RESULTS

**Figure 1:**
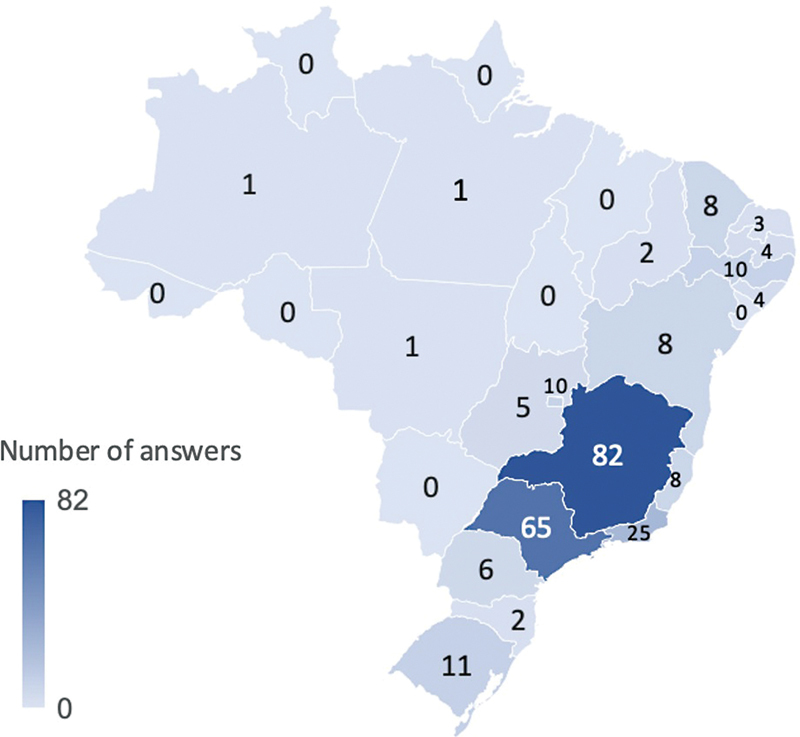
Geographical distribution of respondents. Brazil is a federation of 26 states and one federal district. The country is composed of five geographical regions: North, North East, Center-West, Southeast, and South.

**Figure 2:**
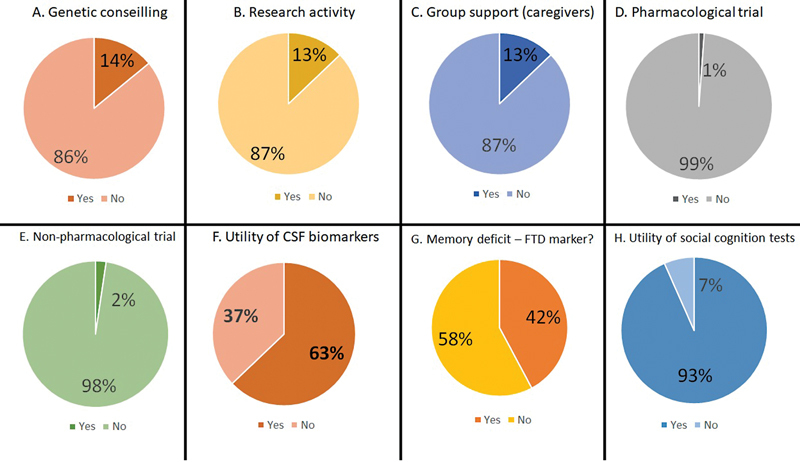
Answers for the following questions: ( **A** ) Does your service offer genetic counselling? ( **B** ) Does your service currently conduct research on frontotemporal dementia (FTD)? ( **C** ) Does your service offer help (support group) for caregivers of patients with FTD? ( **D** ) Has your service ever participated in a Pharmacological Clinical Trial in FTD? ( **E** ) Has your service ever participated in a non-pharmacological Clinical Trial in FTD? ( **F** ) Do you consider the measurement of CSF biomarkers (Abeta, Tau and P-Tau) to be a useful tool in the diagnostic investigation of FTD? ( **G** ) Do you consider episodic memory deficit to be a good marker to differentiate FTD from Alzheimer's disease? ( **H** ) Do you consider that the assessment of functions related to social cognition (recognition of emotions, theory of mind, emotional processing, among others) contributes to improving the diagnosis of FTD?

 Two hundred fifty-six physicians answered the survey. Respondents were from all but eight Brazilian states ( Figure 1 ). The majority of respondents were from Minas Gerais and São Paulo states. Neurologists were the most frequent specialists, followed by geriatricians and psychiatrists ( Table 1 ). Most respondents were set at private clinics, followed by public hospital/clinic and public university hospital ( Table 1 ). Most services do not have databases, do not offer genetic counselling and have no experience in clinical research ( Figure 2 ). 

**Table 1:** Profile of the respondents: characterization of institutions and clinical experience (raw numbers)

*In what type of service do you see patients with frontotemporal dementia (FTD)?*					
Private clinic	Private hospital or clinic	Public hospital or clinic	Private university service	Public university service	
114	21	59	6	56	
*What is your medical specialty?*					
Internal Medicine	Geriatrics	Neurology	Psychiatry	Other	
2	47	163	36	8	
*Which professionals make up the service in which you work?*					
Geriatrician	Neurologist	Neuropsychologist	Occupational therapist	Psychiatrist	Speech therapist
116	201	100	68	129	89
*How many patients with FTD (all variants) do you currently see?*					
< 1/month	1/month	2–10/month	11–20/month	> 20/month	
57	74	112	6	7	
*How many new FTD diagnoses (all variants) do you make per year, approximately?*					
1–5	6–10	11–15	> 15		
177	51	10	18		
*Does your service have a database of patients with FTD?*					
None	Neuroimaging	Clinical and cognitive-behavioral	Clinical	Brain	Biological samples
177	14	45	43	6	5

 The majority of respondents (112/256) reported following 2–10 FTD patients per month; 177/256 reported establishing 1–5 new FTD diagnoses per year ( Table 1 ). Most physicians (224/256) do not follow patients or families with confirmed genetic mutations. The most frequent genetic mutations under clinical follow-up were *c9orf72* ( *n*  = 17), *GRN* ( *n*  = 10) and *MAPT* ( *n*  = 9). The majority of genetic cases are under neurological assistance (23/26). 

 Maniform symptoms, language deficits and family history were the most common aspects investigated in the clinical interview of patients with suspected FTD ( Table 2 ). The majority of respondents reported that they did not use specific tools to investigate behavioral symptoms ( Table 2 ). The Neuropsychiatric Inventory was the most frequent tool to assess behavioral disorders. The Mini-Mental State Exam (MMSE), the Frontal Assessment Battery (FAB) and the Montreal Cognitive Assessment (MoCA) were the most frequent cognitive tools employed by the respondents ( Table 2 ). 

**Table 2:** Clinical procedures for the diagnosis of Frontotemporal dementia [percentages (raw number)]

	Neurologists *(n = 163)*	Psychiatrists *(n = 36)*	Geriatricians *(n = 47)*	General practitioner *(n = 2)*	Other *(n = 8)*	ALL ( *n* = 256)
*What aspects do you usually investigate in the interview of suspected patients?*						
Family history	80.3% (131)	86.1% (31)	87.2% (41)	50% (1)	100% (8)	83% (212)
Orientation deficits	61.3% (100)	47.2% (17)	64.8% (24)	100% (2)	62.5% (5)	58% (148)
Memory disorders	66.8% (109)	63.9% (23)	68% (32)	50% (1)	87.5% (7)	67% (172)
Language deficits	82.2% (134)	94.4% (34)	76.6% (36)	50% (1)	87.5% (7)	83% (212)
Depression	71.1% (116)	72.2% (26)	72.3% (34)	50% (1)	87.5% (7)	72% (184)
Maniform symptoms	80.3% (131)	86.1% (31)	93.6% (44)	50% (1)	87.5% (7)	84% (214)
Learning deficits	34.3% (56)	36.1% (13)	35.1% (17)	50% (1)	37.5% (3)	35% (90)
Behavioral changes	60.1% (98)	100% (36)	0% (0)	0% (0)	100% (8)	55% (142)
*Which neuropsychological tests do you routinely use and/or recommend for investigating suspected cases?*						
ACE-R or ACE-III	9.8% (16)	16.7% (6)	10.6% (5)	0% (0)	12.5% (1)	11% (28)
DRS-MATTIS	19.3% (32)	11.1% (4)	17% (8)	100% (2)	12.5% (1)	18% (47)
MMSE	59.5% (97)	69.4% (25)	80.8% (38)	100% (2)	87.5% (7)	66% (169)
Frontal Assessment Battery	53.4% (87)	61.1% (22)	61.7% (29)	100% (2)	100% (8)	58% (148)
Lexical Fluency	42.3% (69)	44.4% (16)	46.8% (22)	50% (1)	37.5% (3)	43% (111)
Figure Memory Test BCSB	47.2% (77)	41.7% (15)	53.2% (25)	100% (2)	50% (4)	48% (123)
Mini-SEA	6.1% (10)	16.7% (6)	17% (8)	0% (0)	0% (0)	9% (24)
MoCA	54% (88)	80.5% (29)	46.8% (22)	50% (1)	100% (8)	58% (148)
RAVLT	9.2% (15)	25% (9)	10.6% (5)	100% (2)	0% (0)	12% (31)
Stroop	18.4% (30)	19.4% (7)	25.5% (12)	50% (1)	25% (2)	20% (52)
WCST	12.9% (21)	16.7% (6)	8.5% (4)	0% (0)	0% (0)	12% (31)
Other	6.1% (10)	8.3% (3)	2.1% (1)	0% (0)	0% (0)	5.4% (14)
*How do you investigate behavioral changes in suspected patients?*						
CBI	6.1% (10)	2.8% (1)	6.4% (3)	0% (0)	0% (0)	5% (14)
FBI	9.2% (15)	11.1% (4)	12.8% (6)	50% (1)	0% (0)	10% (26)
FTD-FRS	0.6% (1)	2.8% (1)	4.2% (2)	0% (0)	0% (0)	1% (4)
FTLD-CDR	1.8% (3)	0% (0)	4.2% (2)	0% (0)	0% (0)	2% (5)
NPI	37.4% (61)	61.1% (22)	38.3% (18)	100% (2)	37.5% (3)	41% (106)
SAS	5.5% (9)	2.8% (1)	4.2% (2)	0% (0)	0% (0)	5% (12)
Structured interview	3% (50)	27.8% (10)	36.2% (17)	0% (0)	25% (2)	31% (79)
Unstructured interview	68.7% (112)	66.7% (24)	66% (31)	0% (0)	75% (6)	68% (173)
*Which is the biggest difficulty you face in the diagnostic investigation of FTD?*						
Genetic investigation	62.6% (102)	14.9% (7)	32% (15)	50% (1)	0% (0)	49% (125)
CSF biomarkers	11% (18)	11.1% (4)	8.5% (4)	0% (0)	37.5% (3)	11% (29)
Structural neuroimaging (CT or MRI)	3% (5)	0% (0)	6.4% (3)	0% (0)	0% (0)	3% (8)
Functional neuroimaging (SPECT or PET)	11.7% (19)	25% (9)	17% (8)	50% (1)	25% (2)	15% (39)
Formal cognitive assessment	11.7% (19)	34% (16)	36.1% (17)	0% (0)	37.5% (3)	21% (55)

Abbreviations: ACE-III, Addenbrooke Cognitive Evaluation – 3 ^rd^ version; Addenbrooke Cognitive Evaluation – Revised; BCSB, Brief Cognitive Screening Battery; CBI, Cambridge Behavioral Inventory; CSF, cerebrospinal fluid; CT, computed tomography; DRS-MATTIS, Dementia Rating Scale; FBI, Frontal Behavioral Inventory; FTD-FRS, Frontotemporal Dementia Rating Scale; FTLD-CDR, Frontotemporal Lobar Degeneration-Modified Clinical Dementia Rating; Mini-SEA, Short Version of the Social Cognition and Emotional Battery; MMSE, Mini-Mental State Exam; MoCA, Montreal Cognitive Assessment; MRI, magnetic resonance imaging; NPI, Neuropsychiatric Inventory; PET, Positron Emission Tomography; RAVLT, Rey Auditory Verbal Learning Test; SAS, Starkstein Apathy Scale; SPECT, single photon emission computed tomography; WCST, Wisconsin Card Sorting Test.

Trazodone was the most common drug employed in the pharmacological treatment of behavioral disorders associated with FTD, followed by antipsychotics, ASMs, and psychostimulants. Neurologists and geriatricians use trazodone more frequently than psychiatrists; neurologists rarely use psychostimulants, which are more commonly prescribed by geriatricians and psychiatrists. Antipsychotics are similarly used by all specialists. Finally, geriatricians use ASM more frequently than other specialists.

Among the challenges in the diagnostic framework of FTD, most respondents (125/256) reported the difficulty in performing genetic testing as the main limit, followed by difficulties in conducting formal neuropsychological testing (55/256), functional neuroimaging (39/256), CSF biomarkers (29/256) and structural neuroimaging (8/256).

 According to this survey, the three most relevant disorders for the differential diagnosis of FTD are AD ( *n*  = 211), bipolar disorder ( *n*  = 117) and dementia with Lewy bodies ( *n*  = 92) ( Table 3 ). Interestingly, neurologists and psychiatrists did not differ in these responses regarding differential diagnosis. However, geriatricians differed from them, as they ranked late-onset schizophrenia as the third more relevant misdiagnosis, while neurologists and psychiatrists considered dementia with Lewy bodies ( Table 3 ). 

**Table 3:** The main disorders more relevant for differential diagnosis with Frontotemporal dementia (percentages and raw numbers)

	In your opinion, what are the THREE main differential diagnoses of FTD?				
	Neurologists *(n = 163)*	Psychiatrists *(n = 36)*	Geriatricians *(n = 47)*	General practitioner *(n = 2)*	Other *(n = 8)*
Alzheimer's Disease	84% (137)	83.3% (30)	74.4% (35)	100% (2)	87.5% (7)
ADHD	3% (5)	5.5% (2)	2.1% (1)	0% (0)	0% (0)
Corticobasal syndrome	26.4% (43)	22.2% (8)	21.2% (10)	100% (2)	25% (2)
Bipolar disorder	41.7% (68)	63.9% (23)	48.9% (23)	0% (0)	37.5% (3)
Late schizophrenia	22.7% (37)	27.8% (10)	38.3% (18)	0% (0)	25% (2)
Lewy Body Dementia	38.6% (63)	33.3% (12)	29.8% (14)	50% (1)	25% (2)
Major depression disorder	20.2% (33)	13.9% (5)	25.5% (12)	0% (0)	62.5% (5)
OCD	8% (13)	13.9% (5)	8.5% (4)	0% (0)	0% (0)
PSP	19% (31)	8.3% (3)	21.2% (10)	50% (1)	25% (2)
Other primary psychiatric disorder	24.5% (40)	25% (9)	17% (8)	0% (0)	25% (2)
Other disease	0.6% (1)	0% (0)	4.2% (2)	0% (0)	0% (0)

Abbreviations: ADHD, Attention deficit and hyperactivity disorder; FTD, Frontotemporal dementia; OCD, Obsessive-compulsive disorder; PSP, Progressive supranuclear palsy.

 Most respondents (93%) considered that the assessment of social cognition is useful for the diagnosis of FTD, but only 63% considered that AD CSF biomarkers are helpful in the diagnostic procedures ( Figure 2 ). Episodic memory impairment was considered a good marker to differentiate AD from FTD by 42% respondents ( Figure 2 ). 

Regarding the proposals for advancing the knowledge and for improving the assistance of FTD patients in Brazil, the respondents ranked as top 1 priority the creation of a common protocol for the diagnosis and management of FTD patients, followed by the development and validation of new behavioral tools adapted for Brazilian population.

## DISCUSSION

 This is the first effort to investigate current practices in the field of FTD in Brazil. A similar initiative has been conducted in Italy 8 and a previous study collected data from Latin America, mainly from Argentina and Mexico. 9 This survey collected data exclusively from Brazil, thus providing useful information about how FTD patients are managed in the Brazilian scenario, and also providing valuable data to improve the research and the assistance of FTD patients in the country. 

 The present findings do not bring information on the prevalence or the incidence of FTD in Brazil. While the design of the Italian survey 8 enabled the estimation of the total number of cases, our study does not allow this calculation, as it is possible that two or more respondents assist FTD patients at the same center. Therefore, we could overestimate the total number of FTD patients under clinical follow-up in Brazil. 

Most respondents were from the Southeast of Brazil, which is the wealthiest region in the country. This may reflect the unequal distribution of medical specialists across the country. Brazil has a continental territory with marked regional disparities, and the local health services follow this uneven socioeconomic picture. Of note, most respondents were from São Paulo and Minas Gerais states, where there are active research centers dedicated to dementia. This highlights the need to spread new centers and to improve the existing ones across the country, to promote public awareness on dementia, to ameliorate the care of patients, and to facilitate the training of health professionals specialized in dementia care.

 This survey depicts a challenging scenario for the assistance of FTD patients in Brazil. Some responses indicate that medical training on FTD is insufficient among Brazilian physicians. For instance, “behavioural changes,” which are considered an essential feature for the diagnosis of bvFTD, 4 are not commonly inquired on the medical interview of suspected patients. Furthermore, the respondents recommended cognitive tools that are not accurate for the diagnosis of FTD, such as the MMSE and the FAB. These data bring to light the need to improve medical training concerning the assistance to FTD patients. 

 Most respondents (88%) do not follow patients or families with confirmed genetic mutations. Interestingly, most physicians (88%) who follow genetic cases are neurologists. This may be due to the fact that genetic cases usually have earlier onset of symptoms and may present with overlapping syndromes such as motor neuron disease and/or parkinsonism, thus requiring neurological assistance. The *c9orf72* genetic expansion was reported as the most frequent genetic cause of FTD under clinical follow-up, being more common than *GRN* and *MAPT* . Previous data indicated that *GRN* and *MAPT* were the most frequent genetic causes of FTD in two Brazilian reference centers, 10 and *c9orf72 expansion* was present in 7.1% of familial cases in another study. 11 Overall, the few numbers of reported genetic cases in this survey may be explained by the difficulties in performing genetic investigation in Brazil. Indeed, genetic testing is not covered by the public health system, and is available only in research protocols or in private laboratories. According to our survey, only 14% of services offer genetic counselling. As a matter of comparison, genetic analyses along with counselling are available in around half of Italian centers. 8 In line with these difficulties, the majority of respondents (125/256) reported the difficulty in performing genetic investigation as the most relevant limit they face in the diagnostic procedures of FTD. 

 Besides genetic testing, other challenges in the FTD framework were also pointed out by the respondents. For instance, the difficulty to perform formal neuropsychological assessment was the second most frequently reported limitation in FTD diagnostic investigation, being more reported than CSF or neuroimaging investigation. This highlights that the medical assistance in Brazil is limited not only by the lack of advanced technological facilities (e.g., molecular neuroimaging) but also by the scarce number of health professionals specialized in dementia. 2

 The management of behavioral symptoms of FTD is a clinical challenge. There are no specific medications, and physicians usually employ off-label pharmacological treatments. 12 There is evidence of benefit with trazodone, 12 13 and it is frequently used by Brazilian physicians. Similarly, antipsychotics are also commonly employed. ASM and psychostimulants may be used as mood stabilizers and for treatment of apathy, respectively. 12 Interestingly, our results suggest that neurologists prescribe these drugs less frequently than geriatricians and psychiatrists. 

 AD was ranked as the most important diagnosis to be differentiated from FTD, in agreement with a previous Latin-American survey. 9 Surprisingly, only 63% of respondents considered AD CSF biomarkers as a useful tool in the diagnostic framework of FTD. Even if there is no specific biomarker for FTD, CSF biomarkers can accurately differentiate FTD from AD, 14 15 16 which is the main cause of misdiagnosis with FTD. Some reasons may explain the low proportion of physicians who consider AD CSF biomarkers relevant in the diagnostic procedures of FTD. First, the difficulty in performing CSF analyses, as AD biomarkers are expensive and are covered neither by the public health system nor by local medical insurance companies. Moreover, physicians lack experience with CSF markers and there are still methodological issues on biomarkers measurements (e.g., high interlaboratory variability regarding the absolute values of markers), which hamper the widespread use of these tools. The development of CSF biomarkers specific for FTD, and also the perspective of new therapies that will target specific pathophysiological pathways of FTD may change this scenario in the following years. 

 This survey also collected information regarding the perception of how cognitive assessment may help in the diagnosis of FTD. Most physicians (93%) consider that social cognition tests (e.g., theory of mind and facial emotion recognition tests) are useful for the diagnosis. Even if the investigation of social cognition is not formally recommended in consensual criteria of FTD, 4 there is increasing evidence that this investigation provides accurate clinical distinction between bvFTD and AD. 17 18 19 20 Of note, the evaluation of social cognition has been recently recommended to distinguish bvFTD from other primary psychiatric disorders. 21

 On the contrary, the majority of respondents (58%) consider that episodic memory impairment is not a good parameter to differentiate bvFTD from AD. This is in line with increasing evidences showing that episodic memory may be impaired in bvFTD, in a pattern similar to that observed in AD. 22 23

 The creation of a common protocol for the diagnosis and management of FTD patients was ranked as top 1 priority to improve the knowledge and the assistance of FTD patients in Brazil. Importantly, the Brazilian Academy of Neurology recently proposed recommendations for the diagnosis of FTD. 24 This initiative may help to standardize diagnostic procedures across the country. It should also be noted that a FTD Brazilian Research Group ( http://dgp.cnpq.br/dgp/espelhogrupo/308304 ) was created and formally registered at the scientific platform of the Brazilian National Council for scientific and Technological Development (CNPq). This group is open to all researchers on the field and aims to facilitate collaborative research among Brazilian scientists. 

 This survey also shows that research facilities are lacking in the country. Besides the aforementioned difficulties in performing genetic investigation, most centers do not dispose of advanced resources, such as molecular neuroimaging and brain bank. Most of Latin American countries also face these difficulties. 25 Even if there has been a marked increase in the Brazilian scientific production on FTD in recent years, most papers refer to clinical and neuropsychological studies, 26 which have limited impact on the understanding of the underlying pathophysiological basis of FTD. State-of-art research requires appropriate funding for researchers and for improving medical facilities. However, there has been a progressive reduction on Brazilian government investment in scientific research, 27 thus precluding the scientific development of the country. 

The shortcomings of this study should be pointed out. As the invitation to participate was widely diffused through social media, it is not possible to estimate the rate of acceptance of participation. Naturally, there is a sample bias, as those who accept to answer the survey have some familiarity with FTD. Therefore, we are aware that these results are not representative of all physicians in Brazil. We did not collect data about the respondents' medical training. This information is necessary to estimate whether clinical practices vary according to the level of education and experience in the field of FTD. Finally, all data are from the perspective of clinicians, rather than using systems-level data which may provide a more accurate and objective picture of current practice.

The present results highlight the need for education and medical training in diagnostic procedures and in the management of FTD in Brazil. It also highlights the need of structural improvement in advanced facilities for diagnostic and research purposes, as the access to molecular neuroimaging, genetic testing and biological investigation with biomarkers is extremely difficult, even in the few reference centers established in the country. In the perspective of disease-modifying treatments of FTD, it is essential to improve the diagnosis, either by molecular neuroimaging or by genetic tests. We expect that the present study may provide valuable insights for the medical education, clinical training of physicians, and for the development of research in the field of FTD in Brazil.
