# Erratum

**DOI:** 10.1055/s-0044-1779740

**Published:** 2024-02-29

In the article “Current clinical and research practices on frontotemporal dementia in Brazil: a national survey”, with DOI number 10.1055/s-0043-1771173 , published in Arq Neuropsiquiatr. 2023;81:632–640:

In the bibliographical citation of the author Leonardo Cruz de Souza, where it reads:

Souza LC

It should be:

de Souza LC

